# Protective effects of delayed intraventricular TLR7 agonist administration on cerebral white and gray matter following asphyxia in the preterm fetal sheep

**DOI:** 10.1038/s41598-019-45872-y

**Published:** 2019-07-02

**Authors:** Kenta H. T. Cho, Guido Wassink, Robert Galinsky, Bing Xu, Sam Mathai, Simerdeep K. Dhillon, Lotte G. van den Heuij, Joanne O. Davidson, Luke Weaver-Mikaere, Laura Bennet, Alistair J. Gunn, Mhoyra Fraser

**Affiliations:** 1 Department of Physiology, The University of Auckland, Auckland, 1023 New Zealand; 2 The Ritchie Center, Hudson Institute of Medical Research and Department of Obstetrics and Gynaecology, Monash University, Victoria, 3168 Australia; 3 The Tsinghua-Berkeley Shenzhen Institute, Tsinghua University, Shenzhen, 518000 People’s Republic of China

**Keywords:** Hypoxic-ischaemic encephalopathy, Developmental disorders

## Abstract

Preterm brain injury is highly associated with inflammation, which is likely related in part to sterile responses to hypoxia-ischemia. We have recently shown that neuroprotection with inflammatory pre-conditioning in the immature brain is associated with induction of toll-like receptor 7 (TLR7). We therefore tested the hypothesis that central administration of a synthetic TLR7 agonist, gardiquimod (GDQ), after severe hypoxia-ischemia in preterm-equivalent fetal sheep would improve white and gray matter recovery. Fetal sheep at 0.7 of gestation received sham asphyxia or asphyxia induced by umbilical cord occlusion for 25 minutes, followed by a continuous intracerebroventricular infusion of GDQ or vehicle from 1 to 4 hours (total dose 1.8 mg/kg). Sheep were killed 72 hours after asphyxia for histology. GDQ significantly improved survival of immature and mature oligodendrocytes (2′,3′-cyclic-nucleotide 3′-phosphodiesterase, CNPase) and total oligodendrocytes (oligodendrocyte transcription factor 2, Olig-2) within the periventricular and intragyral white matter. There were reduced numbers of cells showing cleaved caspase-3 positive apoptosis and astrogliosis (glial fibrillary acidic protein, GFAP) in both white matter regions. Neuronal survival was increased in the dentate gyrus, caudate and medial thalamic nucleus. Central infusion of GDQ was associated with a robust increase in fetal plasma concentrations of the anti-inflammatory cytokines, interferon-β (IFN-β) and interleukin-10 (IL-10), with no significant change in the concentration of the pro-inflammatory cytokine, tumor necrosis factor-α (TNF-α). In conclusion, delayed administration of the TLR7 agonist, GDQ, after severe hypoxia-ischemia in the developing brain markedly ameliorated white and gray matter damage, in association with upregulation of anti-inflammatory cytokines. These data strongly support the hypothesis that modulation of secondary inflammation may be a viable therapeutic target for injury of the preterm brain.

## Introduction

Preterm infants continue to have a very high risk of adverse neurodevelopmental outcomes^1^. The risk of disability is highly associated with early onset inflammation^2–6^. The link between inflammation and neural injury is complex, but at least in part it is highly likely that inflammation is a sterile response to hypoxia-ischemia^7^.

Following hypoxia-ischemia, dead and dying cells release danger associated molecular patterns (DAMPs), which initiate a downstream pro-inflammatory immune response through activation of pattern recognition receptors (PPRS), such as toll-like receptors (TLRs), on the surface of microglia, astrocytes, brain endothelial cells, and perivascular macrophages^4,8,9^. Consequently, cerebral neuroinflammation is initiated, which occurs through the action of pro-inflammatory cytokines, such as TNF-α, interleukin-6 (IL-6), and interleukin-1β (IL-1β)^4,10–14^. TLRs thus have a reputation for serving a pro-inflammatory function, yet there are alternative TLR signaling pathways^15,16^ that are anti-inflammatory in nature, which may provide a secondary adaptation to attenuate immune responses following acute injury.

A potential candidate is TRL7. TLR7 belongs to a subset of TLRs (including TL3, TLR8 and TLR9) that are associated with the surface membranes of endosomes or lysosomes intracellularly^17^. TLR7 is able to activate the production of type I interferons (IFNs) and inflammatory cytokines, in a myeloid differentiation factor 88 (MyD88)- and interferon regulatory factor 7 (IRF7)-dependent manner^18,19^. TLR7’s ligands include single stranded RNA (ssRNA), and microRNAs (miRNAs), as well as a number of small synthetic drugs including imidazoquinoline family members such as gardiquimod (GDQ)^20–22^.

We have recently shown in preterm fetal sheep that lipopolysaccharide (LPS)-induced preconditioning before hypoxia-ischemia attenuates cellular apoptosis, microglial activation and reactive astrogliosis. These protective responses were associated with up regulation of TLR7 gene expression and central and peripheral upregulation of IFN-β^23^. Moreover, there is evidence that stimulation of TLR7 after stroke in adult mice can reduce injury, and is associated with induction of neuroprotective type I IFNs^24^. TLR7 signaling is also protective against experimental autoimmune encephalomyelitis through IFN-β production and stimulation of IL-10 production and IL-10-inducing cytokines^25–27^.

Of particular interest is the finding that TLR7 is developmentally regulated. In the mouse brain, maximal expression of this potentially protective TLR occurs in late fetal life and the early neonatal period and is upregulated 24 hours following hypoxic-ischemia at postnatal day 9^28,29^. Consequently, TLR7 has potential to improve neurological outcome in the developing brain.

In this study, we examined the hypothesis that central (intracerebroventricular (ICV)) infusion of the TLR7 agonist, GDQ, after acute profound umbilical cord occlusion would reduce white and gray matter damage in 0.7 gestation (term~145 days) preterm fetal sheep. At this age brain maturation is broadly equivalent to human brain development at 28–32 weeks gestation^30^.

## Methods

### Animals and surgical procedures

The University of Auckland Animal Ethics Committee approved the experimental procedures.

Romney–Suffolk cross fetal sheep were instrumented at ~98 days of gestation (gestation ~0.7, term ~ 145 days gestation), equivalent to the human fetus of 28-32 weeks of gestation^30^. The general approach used was similar to that described previously^31,32^. Briefly, ewes were anesthetized by an intravenous injection of propofol (5 mg/kg; AstraZeneca Limited, Auckland, New Zealand), and general anesthesia was maintained using 2–3% isoflurane in O_2_. Ewes received 5 ml of Streptocin (250,000 IU/ml procaine penicillin and 250 mg/ml dihydrostreptomycin; Stockguard Labs, Hamilton, New Zealand) intramuscularly for prophylaxis 30 minutes before the start of surgery. During surgery, maternal fluid balance was maintained with constant saline infusion (250 ml/hour), and the depth of anesthesia, maternal heart rate and respiration in the ewes were constantly monitored.

A maternal midline abdominal incision and uterotomy incision were performed to exteriorize the head, neck, and forelimbs of the fetus. Polyvinyl catheters were placed into the right and left brachial arteries and the right brachial vein of the fetus for pre-ductal blood sampling and arterial blood pressure (mean arterial blood pressure, MAP) measurements. Another polyvinyl catheter was inserted into the amniotic sac by suturing it to the shoulder of the fetus to enable monitoring of intra-amniotic pressure as a reference for fetal blood pressure. An inflatable silicone occluder (OC16HD, 16 mm, *In Vivo* Metric, Healdsburg, CA, USA) was then placed around the umbilical cord. Electroencephalogram (EEG) electrodes were placed on the dura over the parasagittal parietal cortex with a reference electrode placed over the occiput as previously described^23^. A thermistor (IncuTemp-1, Mallinckrodt Pharmaceuticals, Chesterfield, UK) was placed over the parasagittal dura 30 mm anterior to bregma. An ICV catheter was placed into the left lateral ventricle (6 mm anterior and 4 mm lateral to bregma). In the sham occlusion group there was no placement of an ICV cannula. A further pair of electrodes was placed subcutaneously over the right shoulder and chest at apex level and sewn across the chest to measure the fetal electrocardiogram (ECG) as previously described^23^. The head and forelimbs of the fetus were returned to the uterus, the amniotic cavity was filled with warm sterile saline, the uterus subsequently closed, and antibiotics (80 mg Gentamicin, Pharmacia and Upjohn, Rydalmere, New South Wales, Australia) administered into the amniotic sac. The maternal laparotomy skin incision was infiltrated with a local analgesic, 10 ml 0.5% bupivacaine plus adrenaline (AstraZeneca Ltd., Auckland, New Zealand). All electrode leads and polyvinyl catheters were exteriorized via the maternal flank. A polyvinyl catheter was placed in the maternal saphenous vein, to provide access for postoperative maternal care and euthanasia.

### Post-operative care

Following surgery, animals were kept in metabolic cages for the entire period of the study, and placed in 12-hour light and 12-hour dark cycles in a temperature-controlled room (16 ± 1 °C, humidity 50 ± 10%). Ewes were provided with water and food *ad libitum*.

A period of at least 4–5 days recovery was allowed before commencement of experiments. Antibiotics were given daily; 600 mg Crystapen (benzylpenicillin sodium, Novartis, Auckland, New Zealand) and 80 mg Gentamicin (Pharmacia and Upjohn, Perth, Australia) intravenously for 3 days. Fetal and maternal vascular catheters were maintained patent by continuous infusion of heparinized saline (20 U/ml at a rate of 0.15–0.20 ml/hour). Daily fetal and maternal arterial blood samples were collected for measurement of pre-ductal pH, blood gas, base excess (Ciba-Corning Diagnostics 845 blood gas analyzer and cooximeter, Massachusetts, USA), glucose and lactate values (YSI model 2300, Yellow Springs, Ohio, USA) to assess well-being. Only fetuses whose arterial blood gases and lactate measurements were within the normal range (PO_2_ > 17.03 mmHg; pH > 7.32; lactate (<1.2 mmol/l) were included in the experiments.

### Physiological monitoring

Fetal MAP (Novatrans II, MX860; Medex, Hilliard, OH, USA), corrected for maternal movement by subtraction of amniotic fluid pressure, fetal heart rate (FHR) derived from the ECG, and EEG and temperature were recorded continuously from 24 hours before occlusion (102–103 days gestation) until post-mortem (106–107 days gestation) as previously described^23,33^. Data not reported in the present study.

### Experimental protocol

On day 103–104 of gestation, animals were randomly assigned to either sham occlusion (n = 9), occlusion (n = 9) or occlusion + GDQ (n = 7) groups. Fetuses of either sex were included in the study (sham occlusion = 5 female, 4 male; occlusion = 5 female, 4 male; occlusion + GDQ = 4 female, 3 male). Fetal asphyxia was induced by rapid inflation of the umbilical cord for 25 minutes with sterile saline of a defined volume that would provide complete inflation of the occluder and umbilical cord compression as determined in pilot experiments with a transonic flow probe placed around an umbilical vein^34^. This duration of asphyxia is associated with diffuse white matter injury and moderate subcortical neuronal loss^35^, comparable to that observed in preterm infants^36^. Successful establishment of occlusion of the umbilical cord was indicated by a rapid onset of bradycardia with a rise in fetal MAP and by pH and blood gas measurements as previously described^33^.

The TLR7 ligand, GDQ (InvivoGen, San Deigo, CA, USA) was infused into the lateral ventricle of fetuses (occlusion + GDQ group) via an ICV catheter using a CMA-100 microinjection pump (Carnegie Medicin, AB, Stockholm, Sweden). Briefly, fetuses received a primed continuous infusion of 1.8 mg/kg GDQ dissolved in 2 ml of sterile endotoxin-free modified artificial cerebrospinal fluid as previously described^37^ at a rate of 11.1 ul/minute for 3 hours commencing 1 hour after the end of umbilical cord occlusion. This dose is based on evidence of neuroprotection in adult stroke models^24^. Occlusion fetuses received sterile endotoxin-free modified artificial cerebrospinal fluid. There were no significant changes in extradural temperature in either group (sham occlusion, occlusion, occlusion + GDQ) during experimentation (data not shown). Fetal arterial blood samples were collected for measurement of pH, blood gas, base excess, glucose and lactate values before (baseline, −1 hour), during (5 and 17 minutes) and following (10 minutes, 1, 4, 6, 24, 48 and 72 hours) umbilical cord occlusion.

### Post-occlusion and post-mortem

Following occlusion, animals were monitored for a further 3 days and euthanized by intravenous injection of an overdose of pentobarbital sodium (9 g, Pentobarb 300, Chemstock International, Christchurch, New Zealand) for post-mortem examination (day 106–107 of gestation). Fetuses were weighed and sexed. Fetal brains were perfusion fixed *in situ* with 500 ml endotoxin-free heparinized saline followed by 1000 ml of 10% phosphate-buffered formalin, pH 7.4. The fetal brain was removed from the skull and post-fixed in the same fixative for approximately 5 days, then divided into 3 main equivalent blocks (A, B, C) and paraffin embedded using a standard histological procedure. Post-mortem examination and gross histological examination verified proper placement of the ICV catheter. Some local tissue damage was apparent due to ICV catheter placement.

### Histopathology and single-labeling immunocytochemistry

 Coronal sections (A, B, C) of brains collected at post-mortem, were approximately 3–4 mm in thickness and included; the anterior section (A) included the striatum and cortex; the middle section (B) included the thalamus, dorsal horn of the hippocampus and cortex and the posterior section (C) included the thalamus, dorsal and ventral horn of the hippocampus and cortex. Sections were processed and paraffin embedded, then subsequently cut at 10 μm thickness using a microtome passing through the mid-striatum (26 mm anterior to stereotaxic zero) and mid thalamus (17 mm anterior to the stereotaxic zero).

Oven dried and xylene deparaffinized sections were rehydrated in a decreasing alcohol series (100%. 95%, 70%) and then washed with 0.1 mol/l phosphate buffered saline (PBS). Antigen unmasking was performed using citrate buffer (pH 6.0) by the pressure-cooking method (2100 Retriever, Aptum Biologics Ltd, Southampton, UK). Endogenous peroxidase was quenched by incubating the sections with 1% H_2_O_2_ in methanol for 30 minutes in darkness. This method was applied for all antibodies, except Olig-2 in which 1% H_2_O_2_ in PBS was used. Blocking was performed with 3% (vol/vol) normal goat serum ((NGS; Life Technologies Limited, Auckland, NZ) in PBS (NGS-PBS), for 1 hour at room temperature. Washed slides were then incubated with corresponding primary and secondary antibodies overnight in 3% NGS-PBS at 4 °C.

The following primary antibodies were used: reactive microglia were labeled with rabbit monoclonal anti-ionized calcium binding adapter molecule-1 (Iba-1) antibody (1:200, AB178680, RRID:AB_2755129, Abcam, Cambridge, England, UK), reactive astrocytes were labeled with rabbit anti-GFAP (1:500, AB68428, RRID:AB_1209224, Abcam), cells undergoing apoptosis were labeled with rabbit polyclonal anti-cleaved caspase-3, which detects endogenous levels of the large fragment (17/19 kDa) of activated caspase-3 resulting from cleavage adjacent to Asp175 (1:200, 9661, RRID:AB_2341188, Cell Signaling Technology Cleaved Caspase-3 (Asp175), Danvers, MA, USA), immature/mature oligodendrocytes were labeled with mouse monoclonal anti-CNPase (1:200, AB6319, RRID:AB_2082593, Abcam). Rabbit monoclonal anti-Olig-2 (1:200, AB109186, RRID:AB_10861310, Abcam) was used as a marker of all cells in the oligodendrocyte lineage^38^. Neuronal nuclei (NeuN) were labeled with rabbit monoclonal anti-NeuN (1:200, AB177487, Abcam, RRID:AB_2532109) and proliferative cells were labeled with mouse monoclonal anti-Ki-67 (M7240, RRID:AB_2142367, Dako, Sydney, Australia).

Unbound antibody was removed by washing in PBS then incubating overnight with goat anti-mouse biotin-conjugated IgG (CNPase and Ki-67; BA-9200, RRID:AB_2336171, Vector laboratories, California, USA) or 1:200 goat anti-rabbit (Olig-2, NeuN, Iba-1, GFAP and cleaved caspase-3; BA-1000, RRID:AB_2313606, Vector laboratories) in 3% NGS-PBS, at 4 °C. Slides were repeatedly washed in PBS then incubated with 1:200 ExtrAvidin (E2885, Sigma-Aldrich, Auckland, NZ) for 2 hours at room temperature. Sections were treated with diaminobenzidine tetrahydrochloride (DAB, Sigma-Aldrich) to visualize immunoreactivity then washed in PBS and permanently mounted with distyrene plasticizer xylene (DPX, Scharlab, Barcelona, Spain). Negative controls with the absence of primary antibody were run in parallel.

### Immunofluorescence

Brain sections (10 µm) were rehydrated and antigen retrieval performed as described above. Slides were washed with PBS + 0.1% Triton X-100 (PBST) for permeability. Blocking was performed using 10% NGS in PBST for 1 hour at room temperature. For assessment of proliferating cell type, slides were incubated overnight with 1:200 mouse anti-Ki-67 (M7240, Dako Limited) and 1:200 rabbit anti-Olig-2 (AB109186, Abcam) or 1:200 rabbit anti-Iba-1 (AB178680, Abcam) or 1:200 rabbit anti-GFAP (AB68428, Abcam) in 10% NGS-PBST, at 4 °C. Slides were washed and incubated for 2 hours with corresponding fluorescent-labeled anti-rabbit secondary antibody (1:500, Alexa Fluor 488, Molecular Probes, Life Technologies, Carlsbad, CA, USA) or anti-mouse secondary antibody (1:500, Alexa Fluor 568, Molecular Probes) in 10% NGS-PBST, at room temperature. For evaluating microglia polarization, tissue sections were incubated overnight with 1:200 rabbit anti-Iba-1 (AB178680, Abcam) and 1:100 mouse monoclonal anti-human cluster of differentiation 163 (CD163) (MCA1853, RRID:AB_2074540, Bio-Rad, CA, USA) in 10% NGS-PBST, at 4 °C. These were then subsequently incubated for 2 hours with corresponding fluorescent-labeled anti-rabbit secondary antibody (1:500, Alexa Fluor 488, Molecular Probes) or anti-mouse secondary antibody (1:500, Alexa Fluor 568, Molecular Probes) in 10% NGS-PBST, at room temperature. Nucleus counter-staining was performed using 4′,6-diamidino-2-phenylindole (DAPI) (1:10,000, D1306, ThermoFisher, Victoria, Australia). Negative controls with the absence of primary antibody were run in parallel. Slides were then washed and mounted with citifluor (AF1, Citifluor, Hatfield, USA).

### Image analysis and quantification

Regions of the brain used for analysis included the subcortical periventricular white matter (PVWM) and the intragyral white matter (IGWM) tracts, and the mid-striatum (comprising the caudate nucleus and putamen) on sections taken 26 mm anterior to stereotaxic zero. The thalamic regions (medial, MN, and medial geniculate, MGN), the dentate gyrus (DG) and the cornu ammonis (CA) divisions of the dorsal horn of the anterior hippocampus (CA1/2, CA3, CA4) were evaluated from sections taken 17 mm anterior to the stereotaxic zero. Immunopositive cells were imaged using Nikon 80i light microscope (Scitech, Preston, Australia) equipped with DS-Fil-U3 camera at x20 magnification with NIS Elements Br. 4.0 software (Nikon Instruments, Melville, NY, USA) using three fields in the white matter (two IGWM, one PVWM), four fields from the striatum (two caudate nucleus/putamen), two fields of the thalamus (one MN, one MGN) and one field each of the hippocampal divisions (CA1/2, CA3, CA4, DG). Image analysis of immunofluorescence double-labeling was performed using Olympus FV-1000 confocal microscope (Olympus, Tokyo, Japan).

Cell counts of immature and mature oligodendrocytes (CNPase-positive), astrocytes (GFAP-positive) and ramified microglia (Iba-1-positive) were quantified by the appearance of cell bodies with extensive cytoplasmic processes in the white matter tracts. All cells of the oligodendrocyte lineage (Olig-2-positive) and proliferative cells (Ki-67-positive) were quantified with nuclear immunostaining from white matter regions. For assessment of activated microglia, only Iba-1-positive cells presenting with amoeboid morphology with loss of arborizations were counted. NeuN-positive neurons were identified by the presence of normal appearing nuclei; pyknotic cells presenting nuclear condensation or fragmentation were not counted. Cells undergoing apoptosis (cleaved caspase-3-positive) were assessed by the number of cells exhibiting chromatin condensation and nuclear fragmentation from both white and gray matter regions. Immunopositivity was quantified using ImageJ software (National Institutes of Health, USA) by a single assessor blinded to the treatment groups by independent coding of slides. Two sections from each animal including both hemispheres were counted and averaged. Photomicrographs were imaged at x40 magnification.

### Cytokine sample collection and immunoassays

Fetal arterial blood samples were collected for cytokine measurement at the following time-points: (baseline, −1 hour), following (4, 6, 24, 48 and 72 hours) umbilical cord occlusion. The number of time-points assessed were slightly different for IFN-β; this was due to the availability of sufficient sample. To reduce pre-analytic error, blood samples were immediately placed in previously chilled tubes with interior coated spray-dried dipotassium ethylenediaminetetraacetic acid (K_2_EDTA; Vacutainer, Becton Dickinson UK Ltd, Plymouth, UK) to provide the optimum blood/additive ratio, mixed by gentle immersion, centrifuged at 1,500 g for 10 minutes at 4 °C, and stored at −80 °C. On the day of analysis, samples were thawed on ice, gently vortexed, and briefly centrifuged before dispensing into separate wells of enzyme-linked immunosorbent assay (ELISA) plates. All samples analyzed had no signs of hemolysis.

IFN-β fetal plasma levels were determined using a commercially available ovine specific ELISA (BlueGene Biotech, Shanghai, China) according to the manufacturer’s instructions. IFN-β ranged from 0 to 1,000 pg/ml with a detection sensitivity of 1.00 pg/ml. TNF-α and IL-10 concentrations were measured using in-house ELISAs^23,39,40^. TNF-α was detected using antibodies specific to the ovine species (EpitopeTechnologies, Melbourne, Australia). Standards were ovine recombinant TNF-α (Kingfisher Biotech, St. Paul, MN, USA) and ranged from 0 to 10 ng/ml with a detection sensitivity of 0.014 ng/ml. IL-10 was detected using antibodies specific to the bovine species (AbD Serotec, MorphoSys UK Ltd). Standards were recombinant bovine IL-10 (Kingfisher Biotech) and ranged from 0 to 10 ng/ml with a detection sensitivity of 0.009 ng/ml. Internal quality controls were included in all ELISAs and cytokine concentrations were within the detection limit in all samples.

### Data acquisition and statistical analysis

Power calculations to estimate group sizes required were based on observed variance in our similar previous studies of CNPase loss within the PVWM and IGWM regions following occlusion, as well as our preliminary data of the effect of GDQ on CNPase loss^41–43^. Based on this, our group sizes enabled detection of a difference between group means of approximately two standard deviations with a power of 80% and type I error of 5%. Fetal physiological and cytokine measurements were evaluated by analysis of variance (ANOVA), with time as a repeated measure and baseline values as a covariate for split plot analysis. Fisher’s least significant post hoc analysis was used to perform pairwise comparisons when a significant overall effect of group or an interaction between group and time was found. Neuropathological data comparisons between groups were performed using two-way ANOVA (v7.03 GraphPad Software, CA, USA). Where significant mean differences were observed between groups and regions, Bonferroni’s post-hoc test was used for comparisons. If an effect of region and group was found, the effect of group was assessed for each region separately. Linear regression analysis was performed for estimating the relationship between cell density of Olig-2 and caspase-3-positive cells in the occlusion and occlusion + GDQ group. All quantitative data are reported as the mean ± standard error of the mean (SEM). The minimum statistical significance threshold was defined as p < 0.05.

## Results

### Umbilical cord occlusion

There was no difference in the duration of umbilical cord occlusion between occlusion and occlusion + GDQ animals (24.3 ± 0.6 vs. 23.4 ± 0.4 minutes, p > 0.05, mean ± SEM). Umbilical cord occlusion was associated with a significant fall in fetal MAP (sham control, 36.5 ± 0.9 vs. occlusion, 13.8 ± 1.7, and occlusion + GDQ, 10.0 ± 0.6 mmHg, p < 0.05) and FHR (sham control, 189.0 ± 5.0 vs. occlusion, 66.9 ± 2.5, and occlusion + GDQ, 73.2 ± 13.8 bpm, p < 0.05) in both occlusion groups compared to sham occlusion, that were not significantly different between occlusion groups (p > 0.05).

### Fetal arterial blood gas, metabolic status and post-mortem brain weight

Baseline pH, blood gases, glucose, lactate were not different between the three groups (Table 1). Umbilical cord occlusion was associated with marked hypoxemia and metabolic and respiratory acidosis in both occlusion groups (p < 0.05 vs. sham occlusion). Post-occlusion, most variables normalized within a day, but notably plasma glucose values remained elevated in the occlusion + GDQ group until 72 hours compared to both occlusion and sham occlusion groups (p < 0.05). PaO_2_ values were also elevated in both occlusion groups until 72 hours post-occlusion (p < 0.05), while occlusion + GDQ values were significantly greater than those of the occlusion group from 48 to 72 hours (p < 0.05). At 72 hours post-occlusion pH values in the occlusion + GDQ group were significantly lower than sham occlusion (p < 0.05).

**Table 1:** Fetal arterial biochemical parameters (arterial pH, blood gases, glucose and lactate levels) were determined from sham occlusion, occlusion (occlusion + vehicle) and occlusion + GDQ (occlusion + gardiquimod) animals 1 hour prior to the start of 25 minutes of umbilical cord occlusion (baseline), 5 and 17 minutes during occlusion, and 10 minutes, 1, 4, 6, 24, 48 and 72 hours after reperfusion.

Group	Baseline	5 min	17 min	+10 min	+1 h	+4 h	+6 h	+24 h	+48 h	+72 h
***pH***										
Sham Occlusion	7.37 ± 0.01	7.37 ± 0.00	7.37 ± 0.00	7.37 ± 0.01	7.38 ± 0.01	7.37 ± 0.01	7.37 ± 0.01	7.37 ± 0.01	7.37 ± 0.01	7.36 ± 0.01
Occlusion	7.38 ± 0.01	7.04 ± 0.02*	6.85 ± 0.01*	7.16 ± 0.02*	7.29 ± 0.02*	7.39 ± 0.01	7.39 ± 0.01	7.36 ± 0.01	7.36 ± 0.01	7.35 ± 0.01
Occlusion + GDQ	7.37 ± 0.01	7.05 ± 0.02*	6.81 ± 0.01Ф	7.12 ± 0.02 Ф	7.28 ± 0.01*	7.40 ± 0.02	7.38 ± 0.01	7.38 ± 0.01	7.38 ± 0.01	7.39 ± 0.01#
***PaCO***_***2***_ ***(mmHg)***										
Sham Occlusion	48.69 ± 0.99	45.77 ± 1.61	44.99 ± 0.96	45.60 ± 1.13	47.74 ± 0.79	47.29 ± 0.80	48.17 ± 1.02	49.04 ± 0.91	48.46 ± 1.44	48.65 ± 1.07
Occlusion	49.34 ± 1.37	100.09 ± 2.95*	133.68 ± 2.72*	54.62 ± 2.89*	51.18 ± 3.32	47.64 ± 1.93	49.37 ± 1.60	48.26 ± 1.24	46.33 ± 1.78	49.06 ± 1.69
Occlusion + GDQ	46.96 ± 1.38	86.58 ± 4.10Ф	139.30 ± 0.47Ф	52.86 ± 3.50*	47.18 ± 1.30	48.02 ± 1.03	49.56 ± 0.83	44.36 ± 1.64	45.93 ± 1.64	46.13 ± 1.44
***PaO***_***2***_ ***(mmHg)***										
Sham Occlusion	23.2 ± 1.01	22.69 ± 0.65	23.19 ± 0.72	23.56 ± 0.86	23.08 ± 0.95	22.61 ± 0.77	22.67 ± 1.25	22.03 ± 0.86	22.64 ± 0.67	22.99 ± 1.02
Occlusion	24.29 ± 1.28	8.40 ± 0.57*	11.08 ± 0.76*	32.33 ± 1.57*	28.47 ± 1.95*	22.96 ± 1.79	23.33 ± 1.76	25.92 ± 2.01*	26.97 ± 1.99*	27.00 ± 2.00*
Occlusion + GDQ	23.74 ± 0.58	6.48 ± 1.46*	5.15 ± 0.75Ф	34.34 ± 0.88*	30.10 ± 0.62*	24.08 ± 1.98	23.28 ± 0.77	27.24 ± 0.62*	29.90 ± 2.28Ф	30.28 ± 2.10Ф
***Lactate (mmol/L)***										
Sham Occlusion	0.95 ± 0.08	0.90 ± 0.07	0.89 ± 0.08	0.91 ± 0.06	0.93 ± 0.08	0.95 ± 0.08	1.02 ± 0.09	0.89 ± 0.06	0.88 ± 0.08	0.84 ± 0.07
Occlusion	0.89 ± 0.10	4.33 ± 0.33*	7.08 ± 0.25*	6.13 ± 0.40 *	4.39 ± 0.56*	2.85 ± 0.67*	2.53 ± 0.58*	1.09 ± 0.20	0.90 ± 0.12	0.83 ± 0.09
Occlusion + GDQ	0.72 ± 0.07	3.39 ± 0.19*	6.15 ± 0.05*	5.33 ± 0.38 *	4.28 ± 0.51*	2.47 ± 0.66*	3.23 ± 0.44Ф	1.82 ± 0.34Ф	0.73 ± 0.08	0.68 ± 0.08
***Glucose (mmol/L)***										
Sham Occlusion	1.16 ± 0.08	1.12 ± 0.09	1.10 ± 0.10	1.10 ± 0.08	1.16 ± 0.08	1.14 ± 0.11	1.19 ± 0.09	1.16 ± 0.09	1.13 ± 0.07	1.03 ± 0.07
Occlusion	1.01 ± 0.08	0.35 ± 0.03*	0.74 ± 0.06*	1.57 ± 0.05*	1.30 ± 0.09*	1.37 ± 0.12*	1.39 ± 0.08*	1.09 ± 0.09	1.16 ± 0.08	1.07 ± 0.09
Occlusion + GDQ	0.99 ± 0.10	0.37 ± 0.08*	0.78 ± 0.02*	1.66 ± 0.11*	1.66 ± 0.09Ф	1.55 ± 0.07Ф	1.69 ± 0.06Ф	1.97 ± 0.17Ф	1.41 ± 0.09Ф	1.28 ± 0.13Ф

Data are presented as mean ± SEM. *p < 0.05 vs. sham occlusion; ^#^p < 0.05 vs. occlusion; ^Ф^p < 0.05 vs. sham occlusion and occlusion. PaCO_2_: fetal arterial pressure of carbon dioxide; PaO_2_: fetal arterial pressure of oxygen.

At post-mortem, mean brain weight in the occlusion group was significantly reduced compared to sham occlusion (mean ± SEM, occlusion: 25.3 ± 0.7 g; sham occlusion: 29.3 ± 0.9 g, p < 0.01), but did not differ between sham occlusion and occlusion + GDQ (27.7 ± 1.2 g) groups. The ratio brain to body weight in the occlusion group was also significantly reduced (mean ± SEM, occlusion: 15.6 ± 1.27 g/kg; sham occlusion: 19.6 ± 0.7 g/kg, p < 0.02), but did not differ between sham occlusion and occlusion + GDQ (17.0 ± 0.9 g/kg) groups.

### Fetal plasma cytokine concentrations

**Figure 1:**
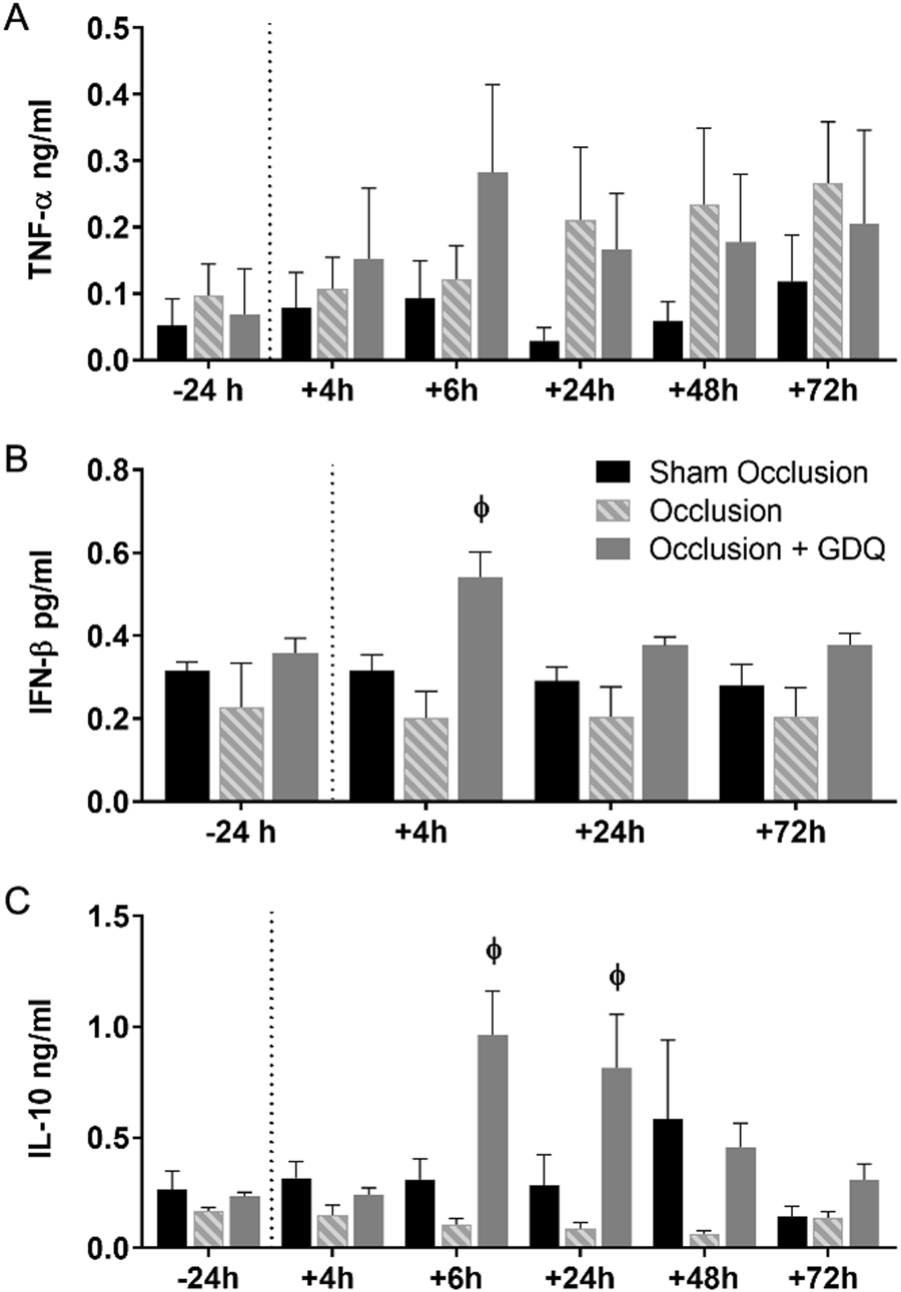
Concentrations of fetal plasma anti-inflammatory cytokines type I IFN-β and IL-10 increased significantly in association with GDQ treatment post-occlusion. Sequential changes in the concentrations of TNF-α (ng/ml), IFN-β (pg/ml) and IL-10 (ng/ml) in sham occlusion, occlusion and occlusion + GDQ animals in relation to the time from the onset of asphyxia are represented in (**A**–**C**), respectively. Dotted line denotes the duration of umbilical occlusion. Data are presented as mean ± SEM. The number of time-points assessed were slightly different for IFN-β; this was due to the availability of sufficient sample. IFN-β: 4 hours, ɸ occlusion + GDQ vs. sham occlusion and occlusion p < 0.01. IL-10: 6 hours + 24 hours, ɸ occlusion + GDQ vs. sham occlusion and occlusion p < 0.001.

In these analyses, our primary objective was to evaluate the impact of acute asphyxia on fetal plasma concentrations of pro-inflammatory and anti-inflammatory cytokines post-occlusion and the possible effects of GDQ treatment. There was no difference in baseline concentrations of the pro-inflammatory cytokine, TNF-α between all three groups (Fig. 1A). Following occlusion, and throughout the remainder of the experiment, there was no significant difference between the three groups for TNF-α. Similarly, there was no difference in baseline fetal plasma concentrations of the anti-inflammatory cytokines, IFN-β and IL-10, between all three groups (Fig. 1B,C). In the occlusion + GDQ group, 3 hours following commencement of ICV administration of GDQ (4 hours post-occlusion), fetal plasma concentrations of IFN-β were significantly higher than sham-occlusion and occlusion groups (p < 0.01, Fig. 1B). Similarly, in the occlusion + GDQ group, concentrations of IL-10 (Fig. 1C) were markedly higher 6 hours post-occlusion compared with sham-occlusion (p < 0.001) and occlusion (p < 0.001) and 24 hours post-occlusion compared to sham-occlusion (p < 0.001) and occlusion (p < 0.001). In the occlusion group, concentrations of IL-10 tended to be less than sham occlusion, but were not significantly different (p > 0.05).

### GDQ reduces oligodendrocyte cell loss and caspase-3 activation

**Figure 2:**
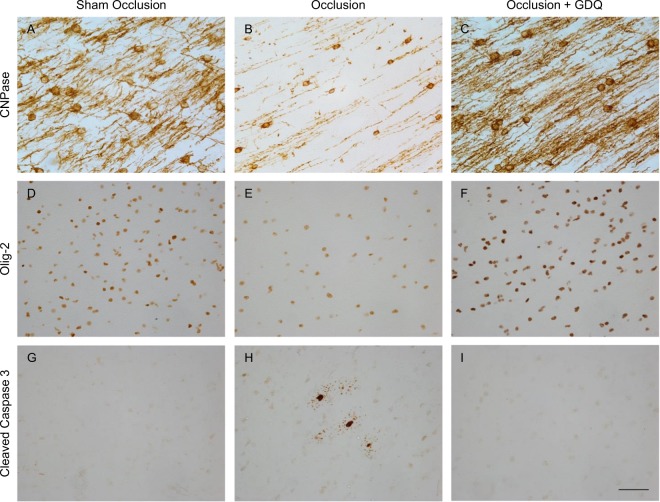
GDQ treatment was associated with increased preservation of the total of oligodendrocyte lineage cells within the periventricular white matter (PVWM) and intragyral white matter (IGWM) regions. Representative photomicrographs of immunohistochemistry for CNPase (**A**–**C**), Oligo-2 (**D**–**F**) and cleaved caspase-3 (**G**–**I**) within the PVWM in sham occlusion, occlusion and occlusion + GDQ groups 72 hours following asphyxia. Representative images for the IGWM regions are not shown. Brains from sham occlusion fetuses exhibited dense CNPAse immunoreactivity within the PVWM (**A**) and IGWM. In the occlusion group, CNPase-positive immature/mature oligodendrocytes exhibited a reduction in cells accompanied by a loss of processes radiating out from the soma (**B**), whereas both cells and processes were restored in the GDQ treated fetuses (**C**). Visual assessment of Olig-2 immunoreactivity confirmed similar robust differences in cell population between sham occlusion (**D**), occlusion (**E**) and occlusion + GDQ groups (**F**). In the occlusion group, PVWM caspase-3 immunopositive cells were detected (**H**). Magnification × 40. Scale bar = 50 μm.

To assess whether GDQ treatment affects oligodendrocyte lineage survival, we first determined the cell density of immature and mature oligodendrocytes within the PVWM and IGWM using the oligodendroglial cell marker CNPase. Qualitative examination of the immunoreactivity of CNPase by light microscopy revealed disorganized and overt reductions in the arborization and number of CNPase-oligodendrocyte processes in the occlusion group within the PVWM and the IGWM (image not shown) relative to sham occlusion (Fig. 2A,B). Strikingly, the morphological appearance of the occlusion + GDQ was similar to that of sham occlusion within the PVWM (Fig. 2C) and IGWM, with increased number of processes and markedly complex arborization.

**Figure 3:**
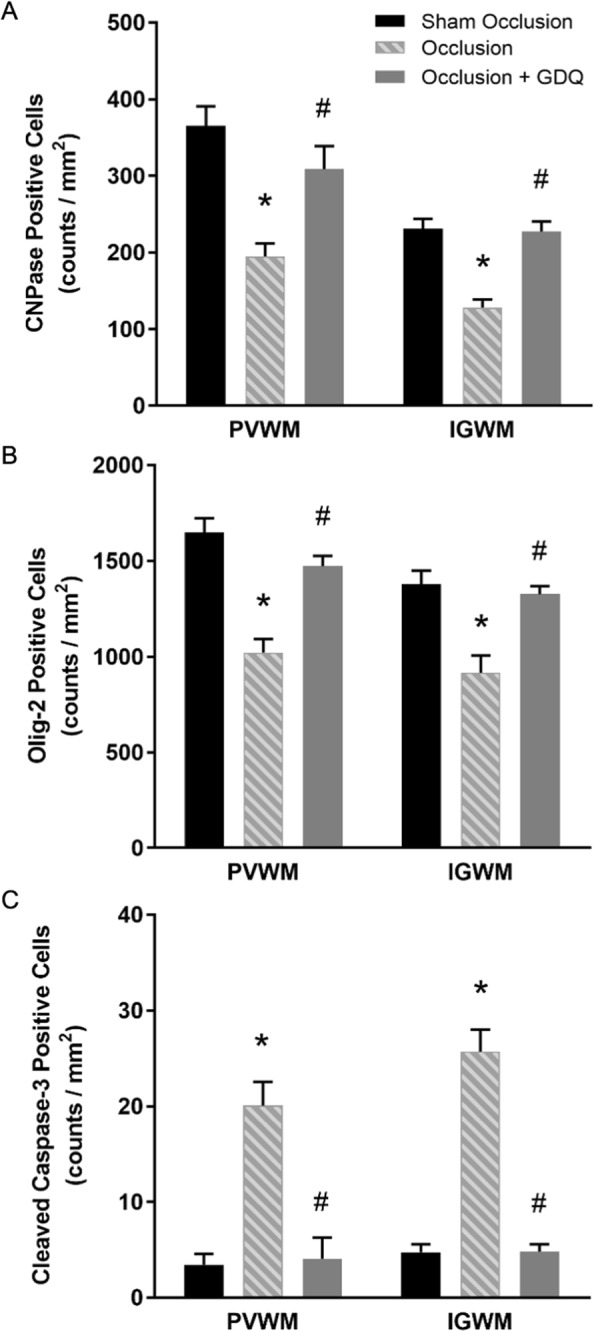
Cell density of oligodendrocytes throughout their lineage within the PVWM and IGWM was restored with GDQ treatment. The figures depict the effect of ICV administration of GDQ on the density of (**A**) CNPase (immature and mature) and (**B**) Oligo-2 (total) oligodendrocyte and (**C**) cleaved caspase-3 immunopositive cells in the PVWM and IGWM after 72 hours of recovery from 25 minutes of umbilical cord occlusion (asphyxia). Cell numbers are mean ± SEM. (**A**) PVWM: *p < 0.001 vs. sham occlusion, ^#^p < 0.001 vs. occlusion. IGWM: *p < 0.001: vs. sham occlusion; ^#^p < 0.002: vs. occlusion. (**B**) PVWM + IGWM: *p < 0.001 vs. sham occlusion; ^#^p < 0.001 vs. occlusion. (**C**) PVWM + IGWM: *p < 0.001 vs. sham occlusion; ^#^p < 0.001 vs. occlusion.

Quantitative assessment confirmed that in the occlusion group, there was a significant reduction in the density of CNPase-positive oligodendrocytes within the PVWM (p < 0.001) and IGWM (p < 0.001) compared to sham occlusion (Fig. 3A). In contrast, the density of CNPase-positive cells in the occlusion + GDQ group was significantly increased in the PVWM (p < 0.001) and IGWM (p < 0.002) compared to occlusion and not significantly different from sham occlusion (Fig. 3A).

Next, we performed an assessment of oligodendrocytes throughout their lineage, including mature oligodendrocytes, using the cell marker Olig-2. As with CNPase, there was a significant reduction in Olig-2 cell density in the occlusion group within both white matter regions compared to sham occlusion (p < 0.001, Fig. 3B; PVWM: Fig. 2D–F, IGWM: image not shown). Likewise, we also found a preservation of Olig-2 cell density with GDQ treatment; in the occlusion + GDQ group cell density was significantly increased in the PVWM (p < 0.001) and IGWM (p < 0.001) compared to occlusion and was no different from sham occlusion (Fig. 3B).

**Figure 4:**
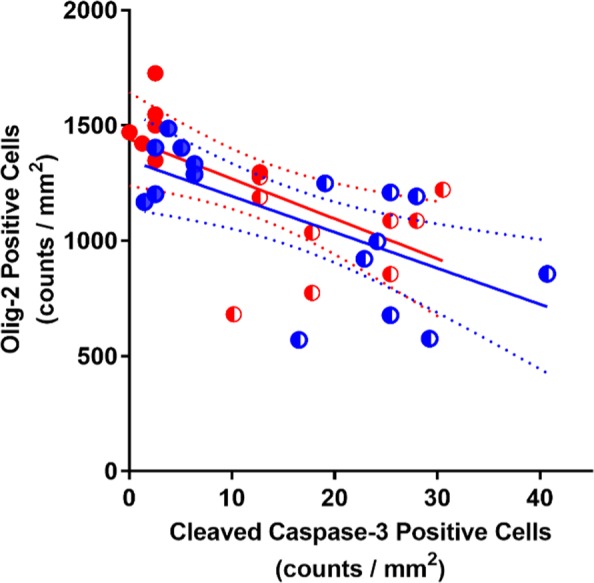
Correlation analysis across all occlusion groups (occlusion and occlusion + GDQ) between the number of Oligo-2 and cleaved caspase-3 cells revealed a significant negative correlation in the PVWM (R^2^ = 0.36, p < 0.03) and IGWM (R^2^ = 0.45, p < 0.01). Dashed lines are the 95% confidence intervals. Full red circle: PVWM occlusion + GDQ; Half red circle: PVWM occlusion; Full blue circle: IGWM occlusion + GDQ; Half blue circle: IGWM occlusion.

Analysis of cell apoptosis using an antibody that specifically recognizes cleaved caspase-3 (activated caspase-3) revealed that in the occlusion group, there was a significant increase in activated caspase-3-positive cells within both white matter regions compared to sham occlusion (p < 0.001, Fig. 3C; PVWM: Fig. 2G–I, IGWM: image not shown). In the occlusion + GDQ group, activated caspase-3 cell density was significantly reduced in both white matter regions compared to occlusion (p < 0.001, Fig. 3C), but not significantly different from sham occlusion. The sham occlusion group exhibited a low degree of developmental apoptosis as measured by a low density of caspase-3-positive cells. Furthermore, we were unable to perform simultaneous double-labeling immunofluorescence with Olig-2 to identify whether these cell types were activated caspase-3-positive as both primary antibodies were from the same species. There was a significant negative correlation between the density of Olig-2 cells and caspase-3-positive cells in both white matter regions (PVWM: R^2^ = 0.36, p < 0.03; IGWM: R^2^ = 0.45, p < 0.01, Fig. 4) of the occlusion + GDQ group, suggesting the increase in Olig-2 density was occurring due to reduced Olig-2 cell death.

### GDQ and oligodendrocyte proliferation

**Figure 5:**
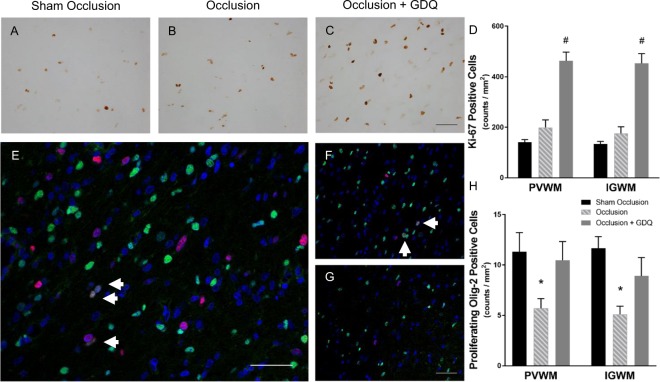
GDQ treatment was associated with increased cell proliferation and restored oligodendrocyte proliferative capability within the white matter. Representative photomicrographs of immunohistochemistry for the proliferative marker Ki-67 within the PVWM in sham occlusion (**A**), occlusion (**B**) and occlusion + GDQ (**C**) 72 hours following asphyxia. Data depict the effect of GDQ on the density of Ki-67-positive cells in the PVWM and IGWM (D, ^#^p < 0.001 vs. sham occlusion and occlusion). Double immunofluorescence labeling with Olig-2 (green) and Ki-67 (red) revealed the proliferative response of Olig-2 positive cells within the PVWM region with GDQ treatment (**E**) was no different from sham occlusion (**F**), whereas it was reduced in the occlusion group (**G**). Dark blue represents DAPI nuclear counterstain. Arrowheads indicate Olig-2/Ki-67-positive staining. Data depict the effect of GDQ on the density of Olig-2/Ki-67-positive cells within the PVWM and IGWM (**H**). PVWM: *p < 0.008 vs. sham occlusion, IGWM: *p < 0.002 vs. sham occlusion. Magnification x 40. Scale bar = 50 µm. Cell numbers are mean ± SEM.

To address the possibility that GDQ treatment was also associated with a compensatory cellular proliferation we undertook single-labeled immunocytochemistry with the proliferative marker Ki-67, which is expressed during the late G1 (Gap1), S (synthesis), G2 (Gap2) and M (mitosis) phases, but not in the resting G0 (Gap0), phase of the cell cycle. Within the PVWM and IGWM, we found no difference in proliferation between sham occlusion and occlusion (Fig. 5D; PVWM: Fig. 5A,B, IGWM: image not shown). In contrast, the occlusion + GDQ group was associated with markedly enhanced proliferative capacity in both white matter regions compared to sham occlusion and occlusion (p < 0.001, Fig. 5C,D). To identify proliferating cells, sections were double-labeled with Olig-2. As illustrated in Fig. 5E–G, a proportion of these cells expressed the proliferation antigen recognized by the Ki-67 antibody in the occlusion + GDQ group. Indeed, the proliferative capacity in both white matter regions of the occlusion + GDQ group was no different from sham occlusion (p = 0.065), whereas it was significantly reduced in the occlusion group (PVWM: p < 0.008, IGWM: p < 0.002, Fig. 5H) when compared to sham occlusion.

### GDQ effects on density and proliferation of reactive astrocytes and activated microglial

**Figure 6:**
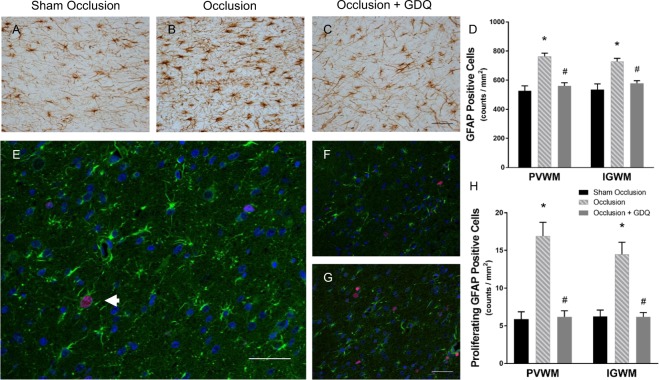
GDQ treatment was associated with reduced density and proliferation of astrocytes. Representative photomicrographs of immunohistochemistry for GFAP, an astrocyte marker, within the PVWM in sham occlusion (**A**), occlusion (**B**) and occlusion + GDQ groups (**C**) 72 hours following asphyxia. Data depict the effect of ICV administration of GDQ on the density of GFAP-positive cells in the PVWM and IGWM (**D**). PVWM: *p < 0.001 vs. sham occlusion, ^#^p < 0.001 vs. occlusion. IGWM: *p < 0.001 vs. sham occlusion; ^#^p < 0.006 vs. occlusion. Double immunofluorescent labeling with GFAP (green) and Ki-67 (red) revealed a marked increase in proliferating astrocytes within the PVWM region in the occlusion group (**E**) compared to sham occlusion (**F**) and occlusion + GDQ groups (**G**). Dark blue represents DAPI nuclear counterstain. Arrowheads indicate GFAP/Ki-67-positive staining. Data depict the effect of ICV administration of GDQ on the density of GFAP/Ki-67-positive cells within the PVWM and IGWM (**H**). PVWM + IGWM: *p < 0.001 vs. sham occlusion, ^#^p < 0.001 vs. occlusion. Magnification x 40. Scale bar = 50 µm. Cell numbers are mean ± SEM.

We next determined whether GDQ treatment was associated with evidence of a concurrent decrease in reactive astrogliosis and microglial activation. It is well established that on activation, astrocytes upregulate the expression of GFAP in a process called gliosis^44^. In agreement with previous findings^43^, significant reactive astrogliosis identified by GFAP-positive astrocytes was observed within the PVWM and IGWM of the occlusion group (p < 0.001, Fig. 6A–C), whereas in the sham occlusion group it was minimal. In the occlusion + GDQ group, astrogliosis was significantly reduced compared to occlusion (p < 0.006, Fig. 6D), but not significantly different from sham occlusion. Subsequent double-labeling immunofluorescence of GFAP-positive astrocytes with Ki-67 antibody (Fig. 6E–G) revealed a marked increase in proliferating astrocyte density within both white matter regions of the occlusion group compared to sham occlusion (p < 0.001, Fig. 6H). In the occlusion + GDQ group, however, the density was substantially reduced compared to occlusion (PVWM: p < 0.001, IGWM: p < 0.001), but not significantly different from sham occlusion. We next determined whether there was an association between the density of oligodendrocytes throughout their lineage and proliferating astrocytes. Results revealed that Olig-2-positive oligodendrocytes were significantly negatively associated with GFAP/Ki-67-positive astrocytes across all groups within the both white matter regions (PVWM: R^2^ = 0.54, p < 0.03; IGWM: R^2^ = 0.47, p < 0.01, data not shown).

**Figure 7:**
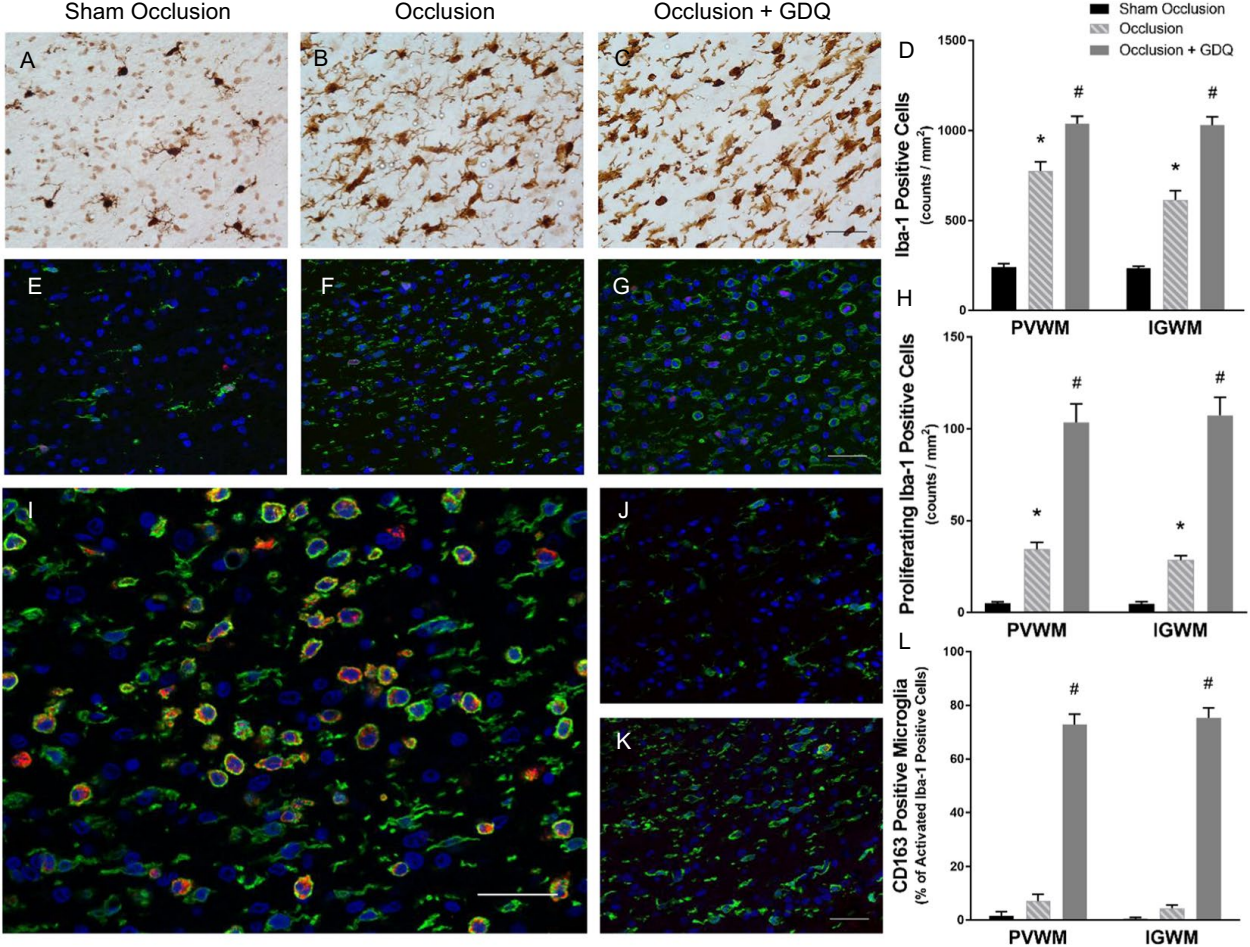
Microglial activation in response to GDQ coincides with increased M2-like polarization. Representative photomicrographs of immunohistochemistry for the microglial marker Iba-1 within the PVWM in sham occlusion (**A**), occlusion (**B**) and occlusion + GDQ (**C**) 72 hours following asphyxia. Data depict the effect of GDQ on the density of Iba-1-positive cells in the PVWM and IGWM (**D**). PVWM + IGWM: *p < 0.001 vs. sham occlusion, ^#^p < 0.001 vs. occlusion. Double immunofluorescent labeling with Iba-1 (green) and Ki-67 (red) revealed a marked increase in proliferating microglial cells within the PVWM in occlusion + GDQ (**G**) compared to sham occlusion (**E**) and occlusion (**F**). Dark blue represents DAPI nuclear counterstain. Data depict the effect of GDQ on the density of Iba-1/Ki-67-positive cells within PVWM and IGWM (**H**). PVWM + IGWM: *p < 0.001 vs. sham occlusion, ^#^p < 0.001 vs. occlusion. Immunolabeling of sections with CD163 and Iba-1 revealed positive reactivity in occlusion + GDQ (**I**) compared to sham occlusion (**J**) and occlusion (**K**). Data represent quantitative analysis of the relative percentages of CD163-expressing amoeboid Iba-1-positive microglia within both white matter regions (L, ^#^P < 0.001 vs. sham occlusion and occlusion). Magnification × 40. Scale bar = 50 µm. Cell numbers are mean ± SEM.

In accordance with clinical evidence of microglial activation in preterm brain injury^45,46^ the density of microglia (Iba-1-positive cells) within the PVWM and IGWM was significantly increased in the occlusion group compared to sham occlusion (p < 0.001, Fig. 7A–D). Unexpectedly, microglial activation in the occlusion + GDQ group far exceeded that of the occlusion group (PVWM: p < 0.001, IGWM: p < 0.001). Furthermore, we identified proliferating microglia by the presence of Ki-67-positive nuclei surrounded by Iba-1-positive cytoplasm (Fig. 7E–G). Quantitative assessment revealed a significant increase in microglial cell proliferation in the occlusion group (PVWM: p < 0.001, IGWM: p < 0.001, Fig. 7H). However, in the occlusion + GDQ group, the proliferative capacity within both white matter regions was markedly greater than occlusion (p < 0.001).

Although morphologically activated microglia generally possess a pro-inflammatory M1 phenotype, we next explored the possibility that the observed microglial proliferation in the occlusion + GDQ group represented polarization towards the anti-inflammatory M2 phenotype. In support of this notion, as illustrated in Fig. 7I, immunolabeling of sections with both cluster of differentiation (CD)-163 (a cell surface antigen, considered to be associated with the M2-like microglia/macrophage phenotype)^47–50^ and Iba-1 antibodies demonstrated that in the occlusion + GDQ group, microglia from both white matter regions showed positive reactivity to CD163 and Iba-1. Further, within these regions quantitative analysis of the relative percentages of CD163-expressing Iba-1-positive microglia revealed significant accumulation of this reparative M2-like microglia within both white matter regions compared to sham occlusion and occlusion groups (p < 0.001, Fig. 7L). Lastly, we were unable to perform a qualitative assessment of M1-like microglial phenotypes due to the lack of cell specificity of markers tested (CD68, CD86, granulocyte-macrophage colony-stimulating factor (GM-CSF) and inducible nitric oxide synthase (iNOS)) for the ovine species.

### GDQ effects on neuronal cell density and caspase-3 activation within hippocampal and selected subcortical regions

**Figure 8:**
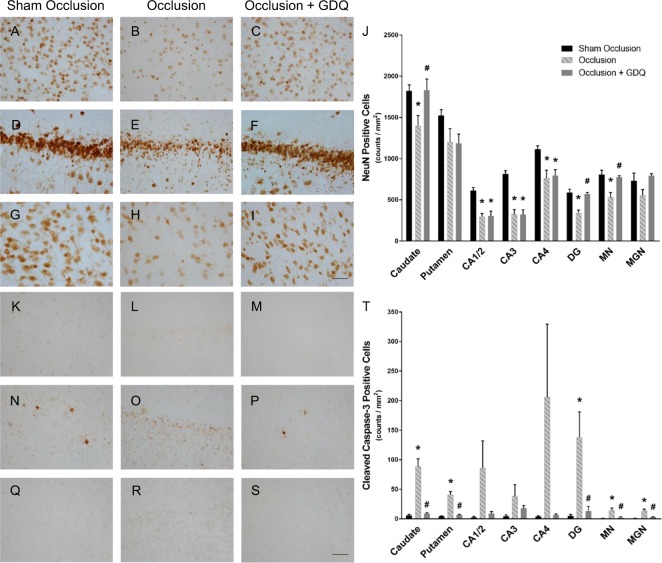
Effects of GDQ on neuronal density and caspase-3 activation within hippocampal and selected subcortical regions. Representative photomicrographs of neurons (NeuN-positive cells, (**A**–**I**) and apoptotic cells (cleaved caspase-3-positive cells, (**K**–**S**), in the striatal caudate nucleus (NeuN: **A**–**C**, caspase-3: **K**–**M**), dentate gyrus (DG, NeuN: **D**–**F**, caspase-3: **N**–**P**) of the hippocampus and thalamic medial nucleus (MN, NeuN: **G**–**I**, caspase-3: **Q**–**S**) in sham occlusion, occlusion and occlusion + GDQ 72 hours following asphyxia. Data depict the effect of GDQ on the density of neuronal (**J**) and caspase-3-positive cells (**T**) within the caudate nucleus, putamen, hippocampal divisions CA1/2, CA3, CA4 and DG, thalamic MN and medial geniculate nucleus (MGN). NeuN: Caudate nucleus: *p < 0.02 vs. sham occlusion, ^#^p < 0.04 vs. occlusion. CA1/2: *p < 0.001 vs. sham occlusion. CA3 *p < 0.001 vs. sham occlusion. CA4: *p < 0.01 vs. sham occlusion. DG: *p < 0.001 vs. sham occlusion, p < 0.002 vs. occlusion. MN: *p < 0.002 vs. sham occlusion, ^#^p < 0.02 vs. occlusion. Caspase-3: *p < 0.005 vs. sham occlusion; ^#^p < 0.005 vs. occlusion. Magnification × 40. Scale bar = 50 µm. Cell numbers are mean ± SEM.

Our studies of subcortical brain structures focused on the hippocampus (CA1/2, CA3, CA4 and DG), basal ganglia (caudate nucleus and putamen) and neighboring medial thalamic nuclei (medial nucleus, MN; medial geniculate nucleus, MGN), because they are most vulnerable in the preterm (Fig. 8)^51^. The occlusion group exhibited a significant decrease in neuronal density, as visualized by NeuN immunoreactivity, within the caudate nucleus (p < 0.02), the CA1/2 (p < 0.001), CA3 (p < 0.001), CA4 (p < 0.01), and DG (p < 0.001) of the hippocampus, and the medial nucleus (p < 0.002, Fig. 8A–I and J). In the occlusion + GDQ group, among the eight regions assessed (Fig. 8J), results showed that neuronal cell density was significantly improved in both the caudate (p < 0.04), DG (p < 0.002) and medial nucleus (p < 0.02) compared to occlusion and was not different from sham occlusion. There was no difference in neuronal density of the putamen and MGN between groups.

Detection of caspase-3-positive apoptotic cells revealed a significantly greater cell density in the caudate, putamen, medial thalamic nuclei (MN, MGN) and DG regions in the occlusion group (p < 0.005, Fig. 8K–S and T) compared to sham occlusion. In the occlusion + GDQ group, activated caspase-3 cell density was significantly reduced in all regions apart from CA1/2, CA3, CA4 hippocampal regions, compared to occlusion (p < 0.005; Fig. 8T), but not significantly different from sham occlusion.

### GDQ effects on proliferation within hippocampal and selected subcortical regions

**Figure 9:**
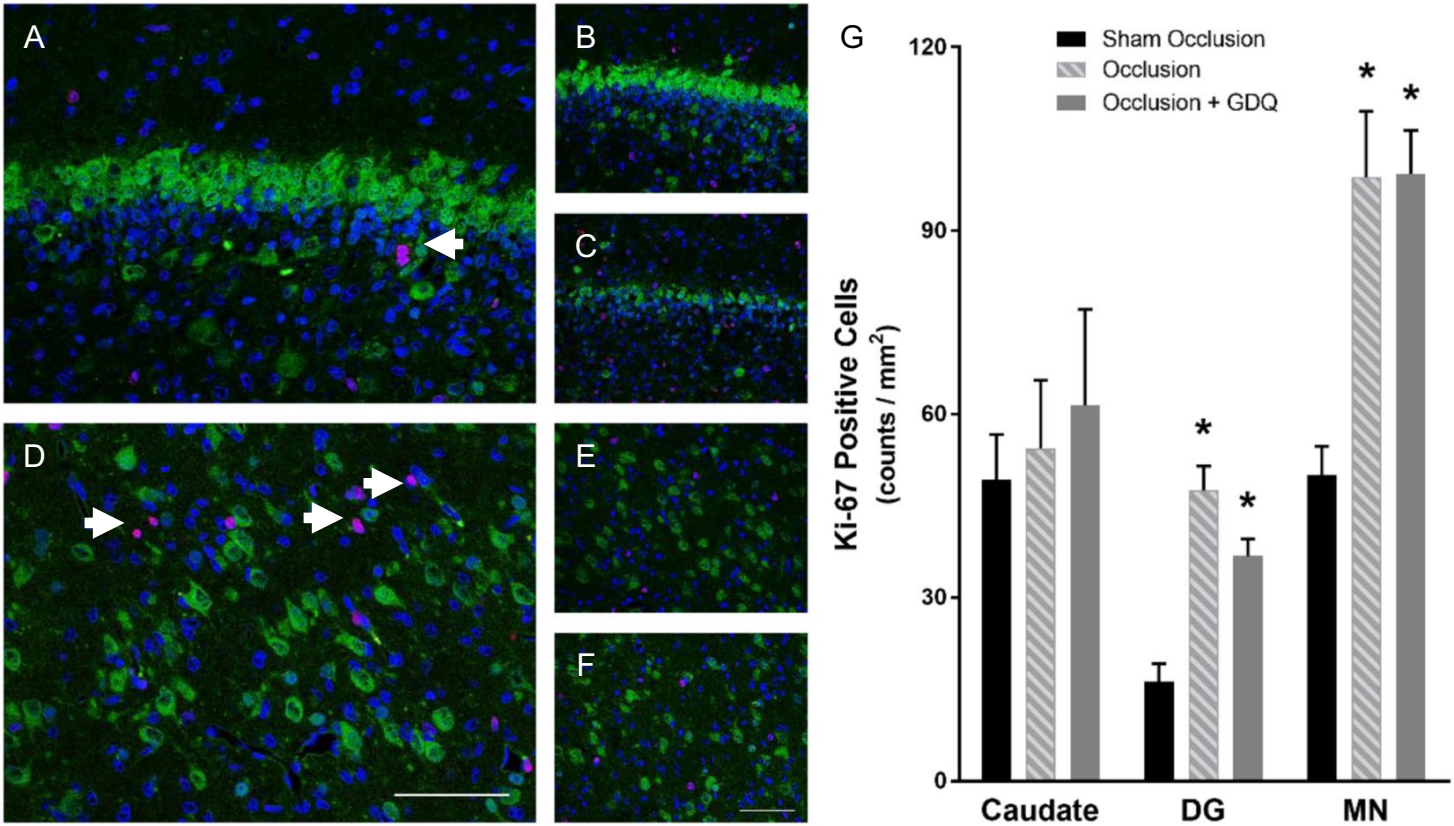
Cell proliferation in the hippocampus and sub-cortical regions. Representative photomicrographs of immunohistochemistry for the neuronal marker, NeuN (green) and the proliferation marker, Ki-67 (red), revealed a proliferative response independent of NeuN-positive cells within the dentate gyrus (DG, **A**–**C**) and thalamic medial nucleus (**D**–**F**) in both occlusion + GDQ (**A**,**D**), and occlusion (**C**,**F**) compared to sham occlusion (**B**,**E**). DG: *p < 0.001 occlusion vs. sham occlusion, *p < 0.01 occlusion + GDQ vs sham occlusion. MN: *p < 0.002 occlusion vs. sham occlusion, p < 0.01 occlusion + GDQ vs. sham occlusion. Magnification × 40. Scale bar = 50 µm. Cell numbers are mean ± SEM.

We next determined the possibility that GDQ treatment was associated with cellular proliferation in the hippocampus and subcortical regions. Among the eight regions assessed, evidence of Ki-67-positive proliferative cells was apparent for both occlusion and occlusion + GDQ groups in only the DG and MN regions (Fig. 9A–F). While the proliferative capacity in both regions was no different between the two occlusion groups, they were both significantly increased compared to sham occlusion (DG: occlusion p < 0.001, occlusion + GDQ p < 0.01; MN: occlusion p < 0.002, occlusion + GDQ p < 0.01 Fig. 9G). Furthermore, Ki-67 immunoreactivity as expected did not colocalize with NeuN, a marker of mature neurons and by definition post-mitotic. Analyses were conducted using doublecortin (DCX), a marker of immature neurons; however, we were unable to generate reliable double-labeling with Ki-67.

## Discussion

In infants born prematurely evidence suggests that loss and subsequent dysmaturation of oligodendrocyte progenitors, is the major driver to myelination failure and alterations to white matter microstructure in later postnatal life^45,52,53^. Currently, there are no effective therapeutic strategies for prevention or amelioration of white matter injury. Our findings document a new approach to treat preterm white matter brain injury using a selective immunomodulatory imidazoquinoline-based TLR7 agonist, GDQ. We demonstrate for the first time that GDQ provides significant neuroprotection in a preterm asphyxia fetal sheep model. GDQ improved survival of immature and mature oligodendrocytes (CNPase) and total oligodendrocytes (Olig-2) within the periventricular and intragyral white matter 72 hours after transient umbilical cord occlusion. Moreover, this occurred in association with a robust reduction in cellular apoptosis (caspase-3) and astrogliosis (GFAP) within both white matter regions.

We were unable to identify the cell type of caspase-3 immunopositive cells. However, we found a significant negative correlation between the density of Olig-2 cells and caspase-3 in both white matter regions suggesting the increase in Olig-2 density was due to a reduction in Olig-2 cell death. Oligodendroctyte proliferation is evoked in response to insults such as hypoxia-ischemia and presumably involves migration of oligodendrocyte progenitors to sites of injury within hours post-injury^54–56^. Thus, we considered the possibility that the increase in Olig-2 cell density could reflect an increase in proliferation. Density of cells double immunolabeled for Ki-67 and Olig-2 revealed a marked reduction in total oligodendrocyte proliferation after occlusion in both white matter regions, whereas there was no difference between sham control and GDQ treatment. Although, there was a trend for increased density approaching, but not reaching significance (p = 0.065) compared to occlusion suggesting GDQ, at least in part, evoked generation of new oligodendrocytes. We acknowledge, however, that in future a thorough examination of GDQ’s effects on the entirety of the oligodendrocyte lineage series including early progenitor cells and pre-oligodendrocytes is required.

In preterm infants, a consequence of white matter injury is reactive gliosis that involves both microglia and astrocytes^45,57^. It is noteworthy that in both white matter regions we observed significantly greater density of Ki-67-positive proliferative microglia with GDQ treatment compared to occlusion. This finding led us to assess whether there was a phenotypic change in microglia. Although whilst fully acknowledging the debated limitations in the usefulness of classification of microglia phenotypes^58,59^, the majority of Iba-1-positive microglia expressed CD163, which is thought to represent acquired deactivation and phagocytosis^60,61^ and thus akin to an M2 anti-inflammatory phenotype. As well, CD163 is strongly induced by anti-inflammatory mediators such as IL-10^62^. The presence of Iba-1/CD163-positive microglia suggests the capability of resident microglia to upregulate CD163. Whether this represents an effect induced by GDQ centrally and/or peripherally remains unknown since Iba-1, a specialized calcium binding protein reportedly specific to microglia^63^ and CD163 both label microglia as well as peripherally derived macrophages^64–66^. Peripheral macrophages can regulate pro-inflammatory signaling pathways in microglia and in turn, reduce inflammatory mediators, such as TNF-α and this interaction represents an important avenue for future study^67^. Lastly, microglia displaying an M1 phenotype are considered to adversely affect oligodendrocyte maturation. We were unable to perform qualitative double-labeling for Iba-1 and markers of M1-like microglial phenotypes due to lack of cell specificity for the ovine species. To resolve the above uncertainties, further studies are required involving distinct microglial gene expression phenotypic markers.

While in the immature brain, astrocytes are thought to play a central neuroprotective role, hypoxemia of sufficient severity can induce numerous pathological processes in astrocytes that compromize both oligodendrocyte cell viability and maturation^3,68,69^. Consistent with this latter process, in both human and preterm fetal sheep, oligodendrocyte progenitor cell proliferation in response to acute degeneration of late oligodendrocyte progenitors, is associated with the degree of astrogliosis^45,70^. Our data demonstrate a significant reduction in both GFAP-positive and GFAP/Ki-67-positive proliferating astrocytes within both white matter regions with GDQ treatment. Moreover, the density of proliferating astrocytes was negatively associated with Olig-2 in both regions of the white matter. Suggesting GDQ potentially improves oligodendrocyte survival through reduced astrogliosis.

Neuronal loss is commonly encountered in preterm infants with damage to cerebral white matter^71,72^. While these abnormalities can potentially occur in any cerebral gray matter region it is more prevalent in subcortical regions^73^. With asphyxia, the combined white and gray matter injury delineated in the present study is consistent with this notion and correlates well with above citations of neuroimaging data. Significant neuronal loss was found within the dorsal caudate nucleus, regions of the dorsal horn of the anterior hippocampus (CA1-4) and dentate gyrus in response to asphyxia. GDQ treatment reduced the extensive loss of neurons within the dentate gyrus, and caudate and medial thalamic nucleus, whereas there was no evidence of improvement in other subcortical regions. An important future direction would be to evaluate whether such favorable effects on neuronal loss translate to beneficial long-term neurological outcomes. A recent study of 4 and 18 days *in vitro* cultured neurons from TLR7 deficient mice highlights the critical role of TLR7 in neuronal development and activity^74^. However, the potential for long-term adverse effects equally requires consideration since exposure of neurons to extracellular let-7 miRNA elicits profound neurodegeneration through TLR7 signaling and activation of caspase-3^75^.

Neuroprotective effects of GDQ may be mediated by anti-inflammatory cytokine actions, although the mechanisms remain unclear. GDQ was associated with a robust increase in fetal plasma concentrations of anti-inflammatory cytokines, IFN-β and IL-10, and no significant change in the pro-inflammatory cytokine, TNF-α. Importantly, the present results confirm GDQ is well-tolerated and is without apparent adverse immune responses. This is consistent with our previous preterm fetal sheep studies of LPS preconditioning of hypoxia-ischemia, whereby low dose LPS prior to acute injury resulted in upregulation of cerebral TLR7 and IFN-β mRNA and protein, and increased fetal plasma IFN-β concentrations in association with decreased cellular apoptosis, and reactive astrogliosis^23^. Also, the aforementioned evidence from adult stroke models suggest neuroprotection against hypoxic-ischemic brain injury can occur through stimulation of TLR7 via GDQ and is associated with induction of neuroprotective type I IFNs^24^. Additionally, TLR7 agonist stimulation in studies of autoimmune encephalomyelitis confers protection through IFN-β production and stimulation of IL-10 production, and IL-10-inducing cytokines^25–27,76^. Finally, we acknowledge that a more in-depth assessment of cytokine profiles is required, since TLR-7 potentially could alter the normal milieu of pro- and anti-inflammatory responses to asphyxia, allowing a damaging bystander effect of inflammation to manifest.

The cellular target through which GDQ mediates neuroprotection *in vivo* requires elucidation. In the present study GDQ was associated with reduced astrogliosis and promotion of an M2-like anti-inflammatory microglial phenotype. As alluded to previously, the latter finding potentially is in response to upregulation of IL-10. Within the immature brain, TLR7 is expressed in microglia^77^. Recent studies of astrocytes and microglia in culture have shown TLR7 agonist stimulation inhibits microglial pro-inflammatory mediator expression and cross-talk with TLR9^78^. Herein, microglia but not astrocytes produce upregulation of TLR7 mRNA expression and anti-inflammatory and anti-apoptotic cytokines in response to TLR7 agonist stimulation and downregulation of TLR4. Remarkably, TLR7 ligands, such as GDQ, also inhibit TLR3 signaling^79^, a potent inducer of cell death of white matter cells (oligodendrocytes)^80,81^ within the periventricular region of the brain. Though the above summation of evidence suggests further possible mechanisms of TLR7-mediated protection, all possibilities remain speculative.

We are unsure as to the mechanistic basis for elevated glucose and lactate levels following completion of GDQ administration. Concerning lactate elevation, the endosomal TLR7 pathway, at least in human blood-derived plasmacytoid dendritic cells, promotes early glycolysis (within minutes following TLR7 stimulation), as supported by elevated rates of lactate efflux^82^. Glycolysis is favored during hypoxia, thus TLR7 stimulation may further enhance this response. However, this does not explain the increase in glucose observed. TLR7 can induce PGE2 and IL-6, known mediators of glucose homeostasis. In addition, central IL-6 enhances glucagon synthesis secretion via catecholaminergic pathways (e.g. epinephrine) by direct action on pancreatic islets^83^, whereas PGE2 can inhibit glucose-induced insulin secretion^84,85^. However, hyperglycemia can have detrimental cerebral effects. Alexandrou *et al*. reported that a prolonged duration of high blood glucose (>8.3 mmol/l) during the first week of life in very preterm infants is associated with reduced white matter volume and associated with poorer motor performance at 2.5 years postnatal age^86^. Nonetheless, experimental evidence can be variably conflicting; for instance, hypothermia can evoke a transient rise in circulating glucose levels in the piglet and near-term fetal sheep, likely through catecholamine release, but is associated with neuroprotection^87,88^.

A translational limitation of our study is that GDQ effects were determined after only 3 hours of continuous infusion commencing 1 hour after acute asphyxia. Ideally, early identification of the onset of injury will allow a therapeutic window, but this is not easily achievable in the clinical setting. Another consideration is that in the preterm infant, white and gray matter injury can progress over days, weeks and months^68,89^. Hence, further investigations are necessary to address the effectiveness of commencing GDQ much later following asphyxia, including whether extending treatment is beneficial or not and to determine effects on normal brain development since TLR7 plays a critical role in the control of neuronal morphology^90^. Finally, successfully translating interventions from preclinical proof of concept to demonstration of therapeutic value in the clinic requires a drug delivery method that is minimally invasive and yet effective. Thus, future studies focusing on non-invasive routes, such as intranasal administration, are required to test GDQ’s effectiveness^91–93^.

## Conclusions

In conclusion, our findings provide the first evidence that cerebral infusion of GDQ, a TLR7 agonist, following asphyxia in the preterm fetal sheep significantly improves survival of immature and mature and total oligodendrocytes within the periventricular and intragyral white matter and subcortical neurons. These improved outcomes occurred in association with decreased cellular apoptosis, and reactive astrogliosis and increased fetal plasma IFN-β and IL-10 concentrations. Future studies are required to elucidate the mechanistic basis of our observations and to extend our findings to assess optimal therapeutic windows for GDQ treatment and long-term outcomes.

### Ethical approval

All experimental procedures were carried out in accordance with protocols approved by The University of Auckland Animal Ethics Committee. This manuscript complies with the Animal Research: Reporting *In Vivo* Experiments (ARRIVE) guidelines, developed by the National Center for the Replacement, Refinement & Reduction of Animals in Research (NC3Rs)^94^.

## Data Availability

The datasets used and/or analyzed during the current study are available from the corresponding author on reasonable request.
